# Examining characteristics of those who receive pedorthic services: A clinical audit

**DOI:** 10.1371/journal.pone.0304443

**Published:** 2024-07-01

**Authors:** Sayed Ahmed, Alex Barwick, Anita Sharma, Md. Zobaer Hasan, Muhammad Ashad Kabir, Susan Nancarrow

**Affiliations:** 1 School of Health and Human Sciences, Southern Cross University, Queensland, Australia; 2 Foot Balance Technology Pty Ltd, Sydney, NSW, Australia; 3 Department of Geriatric Medicine, Nepean Hospital, Penrith, NSW, Australia; 4 Nepean Clinical School, Faculty of Medicine and Health, University of Sydney, Kingswood, NSW, Australia; 5 School of Science, Monash University Malaysia, Bandar Sunway, Selangor Darul Ehsan, Malaysia; 6 School of Computing, Mathematics and Engineering, Charles Sturt University, Bathurst, NSW, Australia; Ningbo University, CHINA

## Abstract

Diabetes-related foot complications, including neuropathic plantar forefoot ulcers, are a significant contributor to morbidity and increased healthcare costs. This retrospective clinical audit examines the characteristics of people accessing pedorthics services who are at risk of neuropathic plantar forefoot ulcer (re)occurrence and the pathways and funding models used to access these services. A clinical record audit was conducted on all patients accessing a pedorthics service who had diabetes and neuropathy with a history of plantar forefoot ulceration. The data included demographics, diabetes and neuropathy duration, main forefoot pathology and other comorbidity, footwear and insole interventions, and health fund access status. A total of 70 patient records were accessed, and relevant data was extracted. The mean age of participants was 64.69 (standard deviation (SD) 11.78) years; 61% were male and 39% female. Duration of diabetes ranged from one to 35 years, with a mean of 14.09 years (SD 6.58). The mean duration of neuropathy was 8.56 (SD 4.16) years. The most common forefoot conditions were bony prominences at 71% (n = 50), rigid flat foot and limited joint mobility (53%, n = 37), and hallux abductovalgus at 47% (n = 33). All participants had hyperkeratosis; 34% (n = 24) had forefoot amputation, and around 34% (n = 24) had a history of digital amputation. Various publicly funded packages and private health insurance were accessed. This study investigates the sociodemographic and medical profiles of individuals with diabetes-related foot complexities prone to neuropathic plantar forefoot ulcers. It is the first to examine patients receiving pedorthic services, informing practitioner surveys and preventive care strategies. Understanding patient characteristics aids in optimising multidisciplinary care and reducing ulcer incidence. Further studies are warranted to explore the field to establish an effective multidisciplinary care approach between medical professionals, podiatrists and pedorthists to optimise patient outcomes.

## Introduction

Diabetes mellitus is a chronic metabolic disorder affecting millions worldwide, with its prevalence continuing to rise. Among the numerous complications associated with diabetes, neuropathic plantar forefoot ulceration is a significant and potentially devastating consequence [1]. Diabetic neuropathy, a common long-term complication of diabetes, makes patients susceptible to insensate foot ulcers, often caused by repetitive pressure and friction due to altered sensation and impaired proprioception [2].

The intricate interplay between diabetes-related foot pathology and biomechanical alterations remains a critical area of investigation in diabetes foot care. Diabetes-related foot pathology frequently manifests in changes to plantar pressure distribution and the development of asymmetrical gait patterns [3]. Understanding these alterations is paramount for elucidating the broader implications of diabetes-related foot complications on gait mechanics and biomechanics.

Neuropathic plantar forefoot ulcers pose substantial challenges for both patients and healthcare providers due to their slow-healing nature and the potential for severe complications, including infection, osteomyelitis, and even lower extremity amputations [4]. Preventing the occurrence of these ulcers is crucial in diabetes management, highlighting the importance of early risk identification and intervention.

Footwear, including custom-made and medical-grade footwear, plays an important role in the prevention of primary and recurrence of ulceration. Diabetes Feet Australia (DFA) guidelines on footwear for people with diabetes recommend prescription guidelines on footwear and insoles design and modifications for effective offloading of the peak plantar pressure at forefoot regions [5–18]. Guidelines also recommend the involvement of footwear specialists such as pedorthists in the management of the at-risk foot [19, 20].

There has been little exploration of the population presenting to such services or the pathways used to access these services. To address the challenges posed by neuropathic plantar forefoot ulcers in patients with diabetes, this research article aims to conduct a clinical audit focusing on patients at high risk of developing these ulcers. In the absence of specific and adequate data for this population group who receive pedorthic services, this study aims to provide the basis of Australian pedorthist survey questionnaire [21] to examine their prescription habit when prescribing devices for this population. Through compiling and analysing pertinent patient data, this study seeks to gain valuable insights into current management practices and identify potential gaps or shortcomings in existing care pathways [21].

## Materials and methods

### Methodological rationale

Clinical audits are a study design commonly used to improve healthcare provision by providing insights into service attributes or practices and identifying potential mechanisms for change [22]. Clinical audits play a pivotal role in improving the quality of healthcare services and patient outcomes [22]. By evaluating current practices, clinical audits identify areas of improvement and guide evidence-based changes in clinical management, thereby enhancing patient care and safety [23]. The attributes examined in this study include the population characteristics such as sociodemographic information, pathologies and comorbidities of those accessing the services, referral pathways, fund providers and eligibility requirements for members referred to pedorthists for the provision of appropriate footwear and insoles. A descriptive analysis of such data gives a localised context to service provision [22].

### Sampling

This retrospective clinical audit sampled a consecutive cohort of all patients who attended a single pedorthic clinic in a metropolitan Australian setting over 18 months to identify the proportion of patients who are at risk of diabetes-related forefoot neuropathic ulceration and their attributes. This particular clinic was chosen due to convenient access to the patient data that was documented through a practice management software system. This clinic receives patient referrals from all around one city.

All the files of patients who were in the area were included in the study if the below inclusion and exclusion criteria in Table 1 were met [24].

**Table 1:** Patient inclusion and exclusion criteria for the retrospective clinical audit study.

Patient inclusion criteria	1. Adults (18+ years old) 2. Diabetes mellitus (T1DM or T2DM) 3. Neuropathy 4. Received offloading intervention
Patient exclusion criteria	1. Ulceration history of the heel, rearfoot or midfoot 2. Charcot neuropathy 3. At risk of rearfoot or midfoot ulcers

The potential participant list included all patients who were presented to the clinic over an 18-month period; the clinical notes were examined to verify the inclusion criteria. Every patient appointment with all pedorthists during the study period was extracted. For patients deemed to meet the inclusion criteria, the relevant patient referral data were extracted from referral forms into the "Audit Data Collection Tool" by using an MS Excel™ spreadsheet. The referrals were in the form of email, a written referral on the clinic pad or a completed structured prescription form [25].

### Tool development

The audit tool is the key element of a clinical audit. The purpose of the audit tool was to gather the required information in a consistent format for data and statistical analysis. In the absence of commonly agreed standards for pedorthic prescription [19], the audit tool used in this study was adopted from commonly used request forms [25] and adapted with stakeholder involvement.

The audit tool was piloted with practitioners and patients during a consultation to ensure all applicable clinical items were covered and mutually understood terminology was used. The resulting adaptations included clinical notes, adherence items and patient preference items. The key information in the tool was sociodemographic, foot morphology, comorbidity, and health fund-related information, and the details are described in the data collection section. The audit tool is available in S1 File.

### Data collection

Data was collected directly into the tool using a Microsoft Excel™ sheet. The data were categorised into three main categories:

1. Sociodemographic: age, gender, body weight, height, country of birth
2. Pathology and comorbidity: duration of diabetes and neuropathy, forefoot pathologies, foot morphology, foot disease outcomes and comorbidity, and
3. Footwear funding body: fund provider’s name.

In the Pedorthic facility in this study, standard clinical practice is to record all clinical information in a clinical practice management software package. Clinical data are recorded from a combination of sources, including self-reports by the patients, general practitioner’s (GP) referral notes with a medical history and current medications, and allied health practitioner referrals that have specific notes on medical conditions and comorbidity with the expected clinical outcome from the pedorthic interventions. The patient data were de-identified before analysis (during extraction).

### Data analysis

The data were tabulated, and then descriptive analyses were performed. The analysed data contained patients’ demographic information, main pathology, comorbidity, footwear fund type and cultural diversity. Specifically, the mean and standard deviation of the continuous variables of age, duration of diabetes, duration of neuropathy, weight, height and BMI were calculated. Frequency counts and percentage of categorical variables, such as diabetes type, gender, presence of deformities, foot morphology, comorbidity, birthplace, and funding, were calculated.

The Pearson Chi-Square Test and Fisher Exact Test (if the assumption of the Chi-Square test is violated) were used to examine the association between the demographic variable (gender) forefoot pathology characteristics (HAV, hammer toe, claw toe, partial amputation, forefoot amputation, forefoot ulcer, bony prominence, flexible flatfoot, rigid flatfoot, limited joint mobility, and cavus foot). Also, we have examined the association between the different co-morbidities (RA, PVD/PAD, lymphoedema, and PTD) and the characteristics of the forefoot pathology.

### Ethical considerations and approvals

Southern Cross University Health and Human Research Ethics Committee gave the Ethics approval for this study, and the Approval number is 2020/028.

The ethical considerations for this study are handling personal data and identifying clinical records.

Participants’ privacy was maintained through de-identification by the respective practitioner (pedorthist), other than the researcher. The clinical data input was provided by treating pedorthists, and there was no risk for the participant relating to clinician data or anonymity. The participant data were de-identified by the treating pedorthists; there was no risk for the participants in relation to data identification.

Data were summarised to ensure that no individual participant was identifiable.

## Results

A total of 421 adults with diabetes and neuropathy who were at risk of plantar foot ulceration and re-ulceration received pedorthic services for foot plantar pressure offloading. Seventy patients met the inclusion criteria.

### Participant characteristics

**Fig 1:**
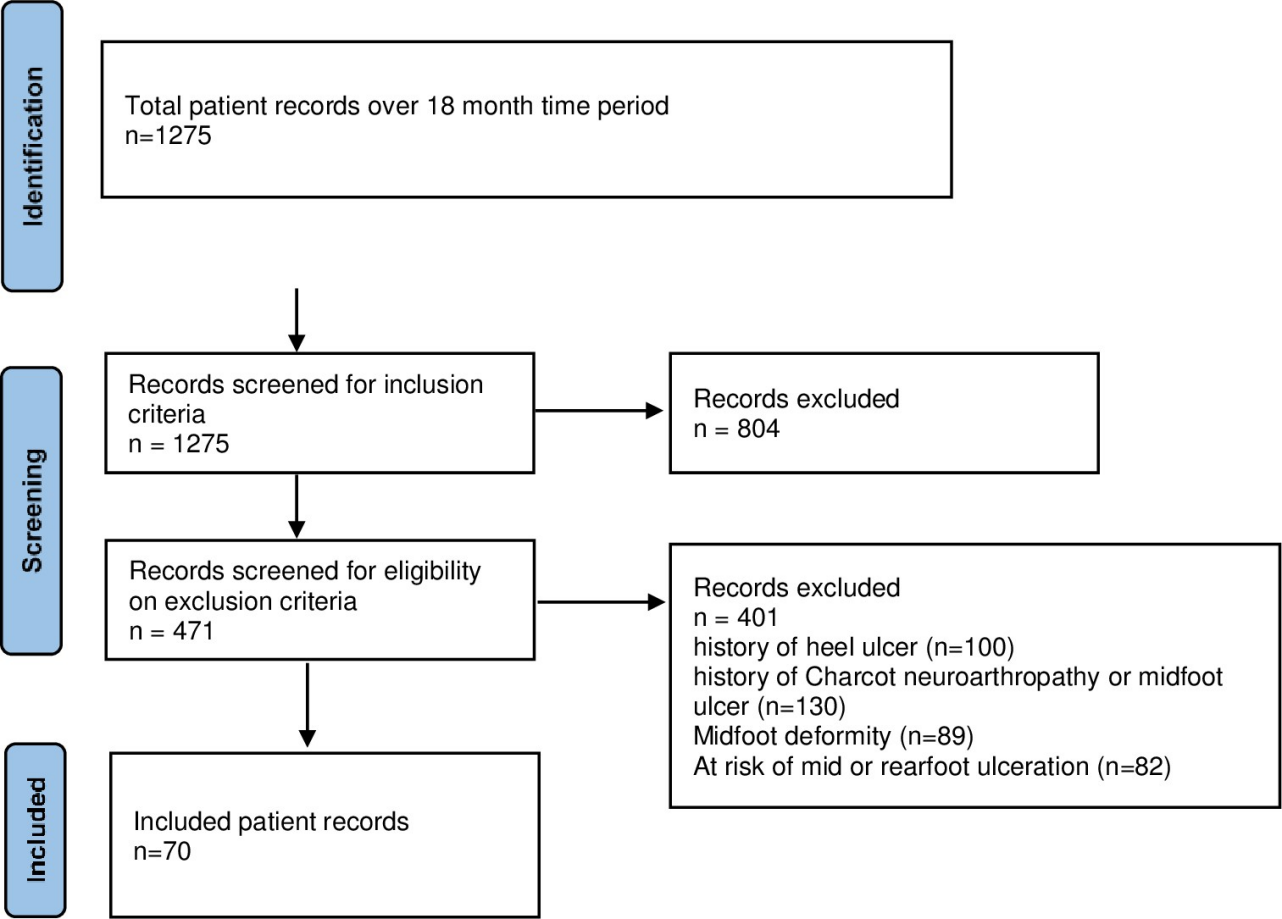
Participant inclusion flowchart.

Fig 1 provides the systematic approach of participant inclusion for this clinical audit study.

Seventy participants (patient records) were included in this study.

The mean age of participants was 64.69 (SD 11.78) years, ranging from 27 to 90 years old. Eighty per cent of participants were between 50 and 80 years of age. A total of 43 males (61.4%) and 27 females (38.6%) with diabetes were included in this study. All participants were overweight to obese, with a mean weight (kg) of 91.37 (SD 14.73), while the mean height (cm) was 171.7 (SD 8.85), ranging from 69kg to 140kg and 152cm to 192cm. The average BMI was 30.96 (SD 4.15).

Most (97.2%) participants had Type-2 diabetes mellitus (T2DM), and only a few (2.8%) had type-1 DM (T1DM). Australia was the birthplace of the highest number of participants (n = 28), followed by England (n = 11), China (n = 5), Fiji (n = 4), Germany and Lebanon (n = 3) each. About 5.7% (n = 4) were of Aboriginal or Torres Strait Islander origin. Most participants (n = 42) were born outside of Australia.

The duration of diabetes among the participants ranges from one to 35 years, with a mean of 14.09 years (SD 6.58). The median was 12 years. Categorically organised data revealed the highest number of participants (n = 25) had diabetes for 11–15 years, followed by six to ten years (n = 21), 21–25 years (n = 10), and 16–20 years (n = 9).

The mean duration of neuropathy was 8.56 (SD 4.16) years, and the median duration of neuropathy was eight years. When categorised into five-year intervals, six to ten years duration of neuropathy is the highest percentage (51.4%) among the participants, followed by 22.9% for 11–15 years duration, 11.4% for 16–20 years duration and 14.3% for the 1–5 years duration.

**Fig 2:**
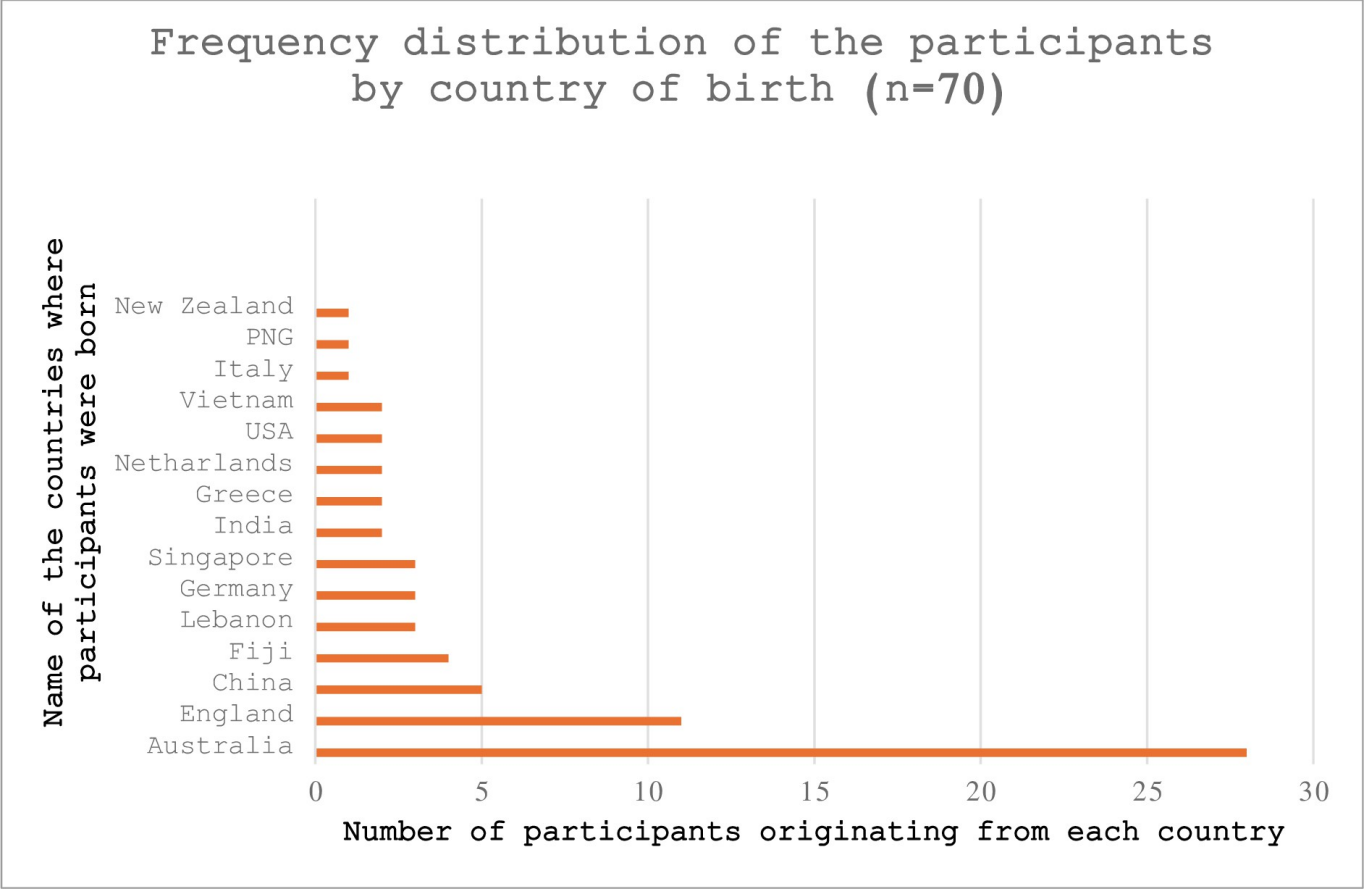
Frequency distribution of the participants by country of birth.

**Fig 3:**
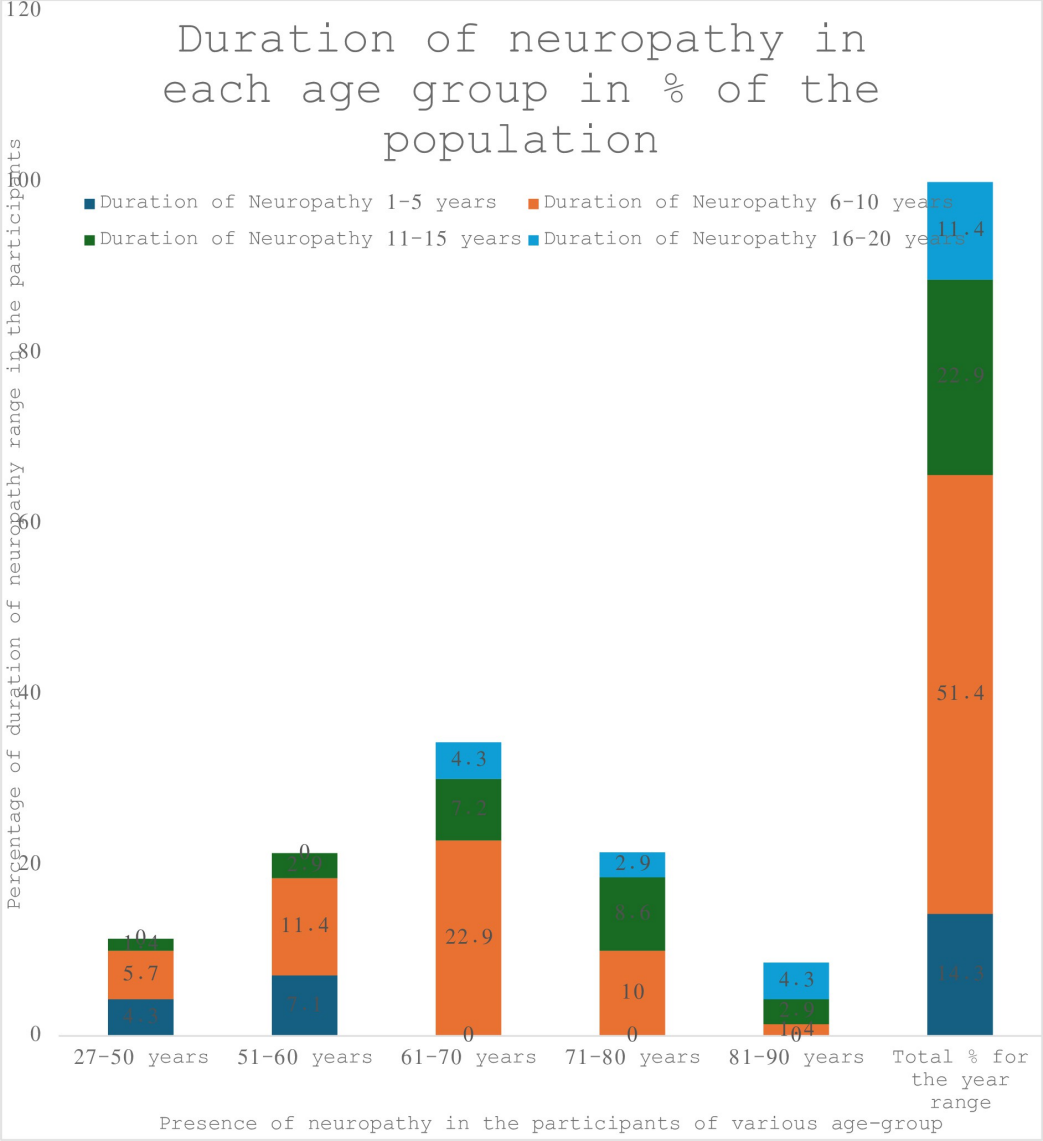
Frequency distribution of duration of neuropathy for each age group in % of the population.

Key demographics, as analysed by age group, are outlined by age group in Tables 2–4 and Figs 2 and 3.

**Table 2:** Percentage distribution of the diabetes participants by BMI within age groups (n = 70).

Age Categories	BMI (kg/m^2^)				Total
	16–18.50 (%)	18.51–25.00 (%)	25.01–30.00 (%)	>30.00 (%)	
25–50 years	-	-	5.7	5.7	11.4
51–60 years	-	1.4	5.7	14.3	21.4
61–65 years	-	2.9	5.7	10	18.6
66–70 years	-	-	10	8.6	18.6
71–80 years	-	2.9	5.7	12.9	21.5
81–90 years	-	-	1.4	7.1	8.5
Total	0	7.2	34.2	58.6	100

**Table 3:** Percentage distribution of the participants by type of diabetes within age groups (n = 70).

Age categories	Diabetes type		Total
	T1DM (%)	T2DM (%)	
27–50 years	1.4	10	11.4
51–60 years	1.4	20	21.4
61–65 years	-	18.6	18.6
66–70 years	-	18.6	18.6
71–80 years	-	21.4	21.4
81–90 years	-	8.6	8.6
Total	2.8	97.2	100.0

**Table 4:** Distribution of diabetes duration by age groups.

Age Categories	Duration of Diabetes					Total n (%)
	1–5 years n (%)	6–10 years n (%)	11–15 years n (%)	16–20 years n (%)	>20 years n (%)	
27–50 years	2	3	3	-	-	8
	(2.86)	(4.29)	(4.29)			(11.4)
51–60 years	-	9	5	1	-	15
		(12.86)	(7.15)	(1.42)		(21.4)
61–65 years	-	-	9	2	3	14
			(12.86)	(2.86)	(4.29)	(20)
66–70 years	-	3	5	2	1	11
		(4.29)	(7.15)	(2.86)	(1.42)	(15.7)
71–80 years	-	5	2	3	6	16
		(7.15)	(2.86)	(4.29)	(8.57)	(22.85)
81–90 years	-	1	1	1	3	6
		(1.42)	(1.42)	(1.42)	(4.29)	(8.57)
Total participants within a range of duration of diabetes	2	21	25	9	10	70
	(2.86)	(30.01)	(35.73)	(12.85)	(18.6)	(100)

Table 5 shows that a significant association exists between gender and three forefoot pathologies—hammer toe, claw toe, and forefoot amputation. Additionally, there is a significant association between RA and four forefoot pathologies—HAV, claw toe, forefoot ulcer, and flexible flatfoot. Other important findings include a significant association between PVD/PAD and two forefoot pathologies, namely partial amputation and forefoot amputation. Furthermore, a significant association exists between PTD and two forefoot pathologies—limited joint mobility and cavus foot.

**Table 5:** Association between [gender & comorbidities] and forefoot pathology characteristics.

	HAV	Hammer Toe	Claw Toe	Partial Amputation	Forefoot Amputation	Forefoot Ulcer	Bony Prominence	Flexible Flatfoot	Rigid Flatfoot	Limited Joint Mobility	Cavus Foot
**Gender**	0.128^p^	3.272^p^*	4.096^p^**	0.003^p^	3.544^f^*	0.189^p^	0.066^p^	1.968^f^	0.074^p^	1.248^p^	1.701^p^
	(0.720)	(0.070)	(0.043)	(0.957)	(0.060)	(0.663)	(0.797)	(0.161)	0.786	(0.264)	(0.192)
**RA**	3.579^p^*	0.484^p^	2.914^p^*	0.667^p^	1.556^f^	4.275^p^**	0.006^p^	5.642^f^**	0.006^p^	0.939^p^	0.484^p^
	(0.059)	(0.487)	(0.088)	(0.414)	(0.212)	(0.039)	(0.939)	(0.018)	(0.939)	(0.269)	(0.487)
**PVD/PAD**	0.026^p^	0.165^p^	0.578^p^	42.414^p^***	6.286^f^**	2.002^p^	0.004^p^	0.085^f^	0.214^p^	0.107^p^	0.349^p^
	(0.873)	(0.685)	(0.447)	(<0.001)	(0.012)	(0.157)	(0.952)	(0.771)	(0.643)	(0.744)	(0.554)
**Lymphodema**	0.129^p^	0.060^p^	0.065^f^	0.612^f^	1.944^f^	0.515^p^	0.149^f^	0.784^f^	0.066^f^	0.129^p^	0.060^p^
	(0.719)	(0.806)	(0.799)	(0.434)	(0.163)	(0.473)	(0.699)	(0.376)	(0.797)	(0.719)	(0.806)
**PTD**	0.079^p^	1.191^p^	0.400^p^	0.057^p^	1.197^f^	0.473^p^	0.150^p^	1.085^f^	1.905^p^	21.611^p^***	15.215^p^***
	(0.778)	(0.275)	(0.527)	(0.811)	(0.274)	(0.492)	(0.699)	(0.298)	(0.168)	(<0.001)	(<0.001)

Note

^***^(p-value<0.*01)*

^**^(p-value<0.*05)*

^*^(*p-value<0*.*10)*

^p:^ Pearson Chi-Square Test

^f:^ Fisher Exact Test.

### Funding providers

**Fig 4:**
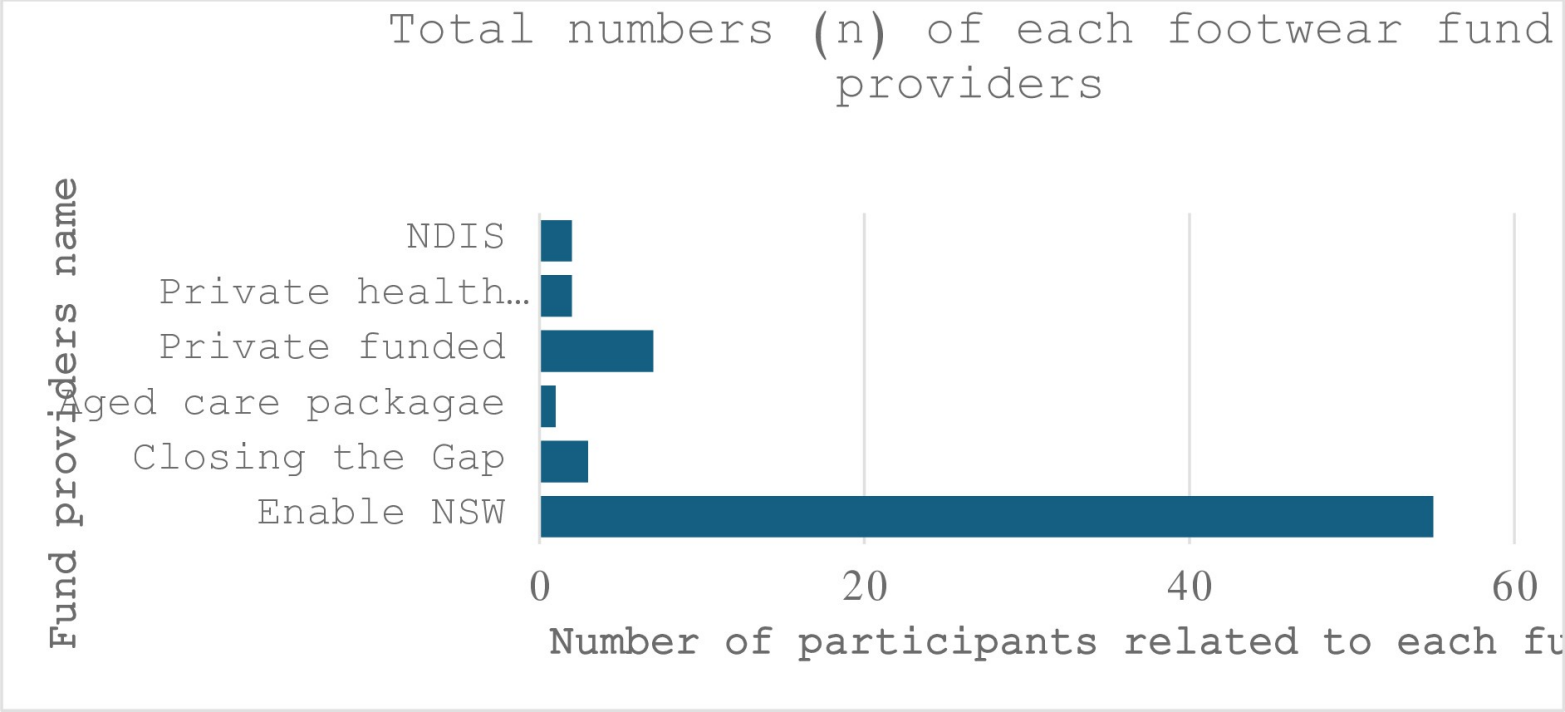
Frequency distribution of footwear fund providers.

As shown in Fig 4, various fund providers fund the footwear in this population group; about 78% (n = 55) were funded by Enable NSW (means tested for NSW residents who meet clinical criteria), followed by privately funded at 10% (n = 7), Closing the Gap (designed for Aboriginal Australians and Torres Strait Islanders) at 4.3% (n = 3), private health insurance 2.9% (n = 2), and aged care package 1.4% (n = 1). The pedorthic practice was based in New South Wales, and the participants’ sociodemographic conditions and medical conditions made them more readily eligible for Enable NSW health fund access. Hence, the number of NSW Health-funded participants is higher in this study. Other states in Australia have similar fund support for those population groups.

### Foot morphology and foot pathology

**Fig 5:**
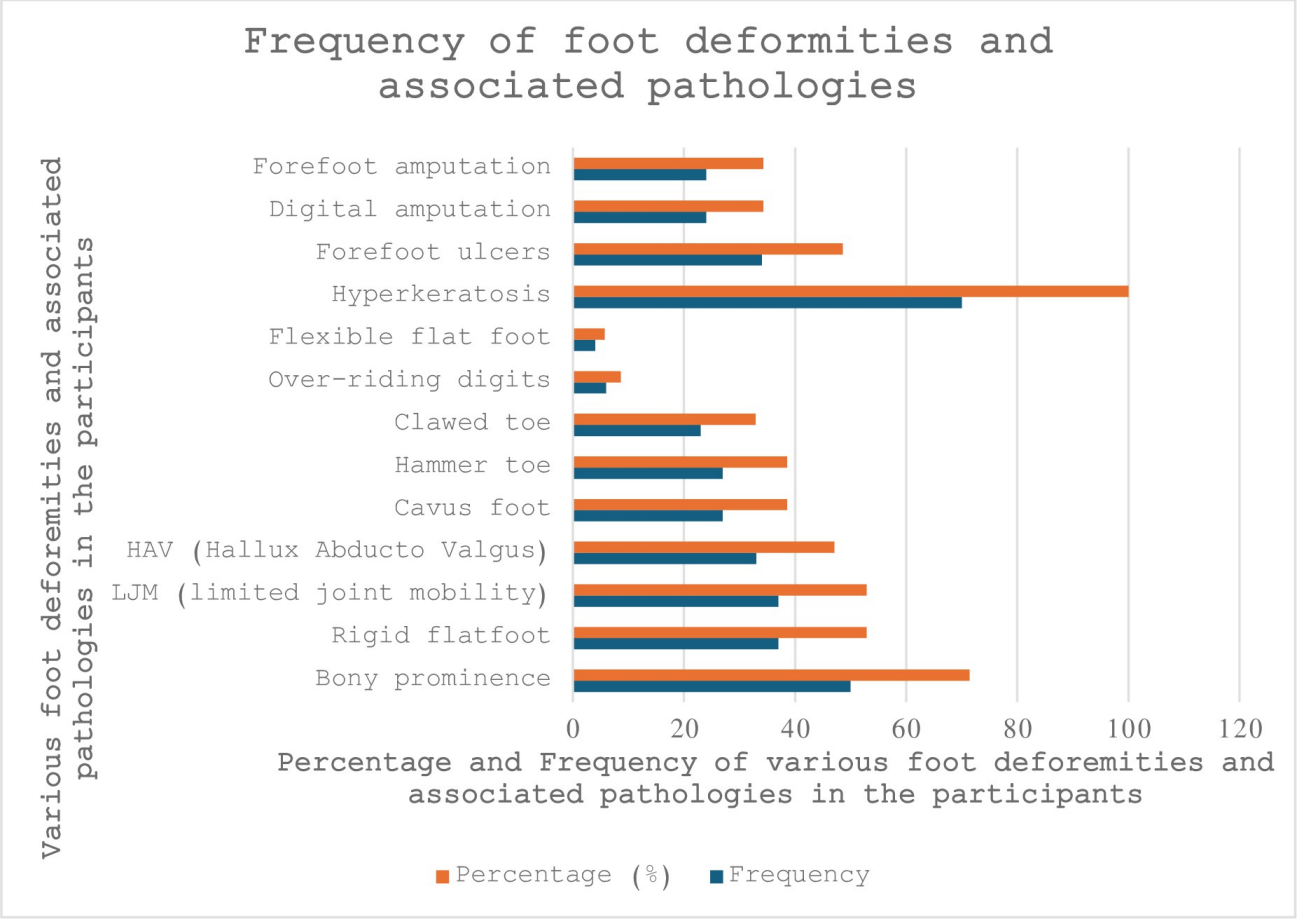
Frequency of foot deformities and associated pathologies.

The foot morphology and foot pathology of the participants in the clinical audit are outlined in Fig 5.

Common recorded conditions were bony prominence at 71% (n = 50) of respondents; rigid flat foot and limited joint mobility (LJM) (53%, n = 37); approximately 47% (n = 33) of participants had HAV; 39% (n = 27) participants had hammertoe and cavus foot conditions, and 33% (n = 23) of participants had clawed toes. Fewer than 9% and 6% had overriding digits and flexible flatfoot, respectively.

Of the foot disease outcomes, hyperkeratosis was the most common condition in the participant group, and everyone (n = 70) had this condition. About half (47%) of the participants had a history of forefoot ulceration. Around one-third, 34% (n = 24) of participants, had forefoot amputation (trans met), and around 34% (n = 24) had a history of digital amputation (partial or single toe or <5 toes).

### Comorbidities

**Fig 6:**
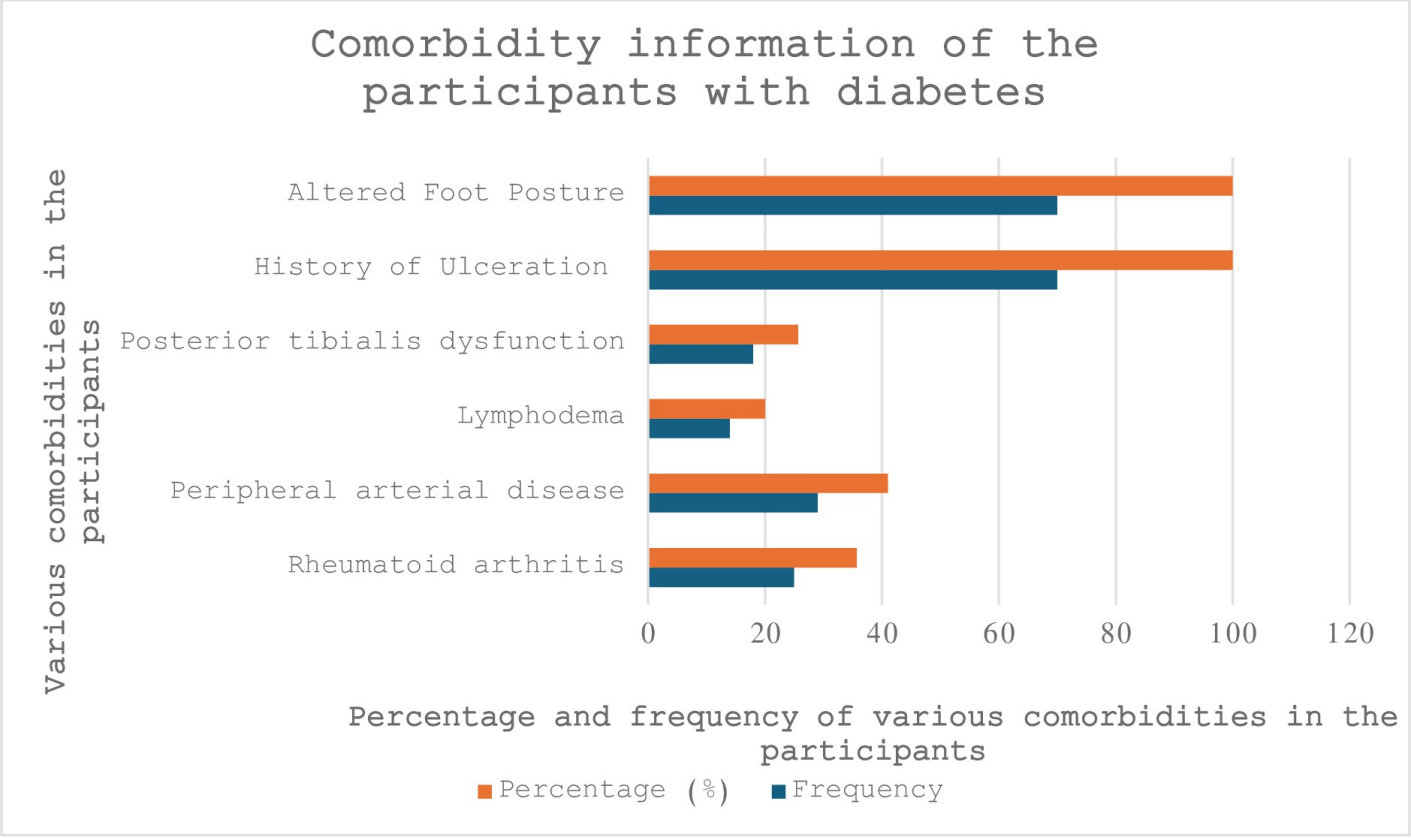
Comorbidity information of the participants with diabetes.

Various comorbidities are common in this group of participants, and the predominant comorbidities in these studied populations are rheumatoid arthritis (RA) 36%, PVD 41%, lymphodema 20%, and posterior tibialis tendon dysfunction (PTTD) 26%. Detailed information is presented in Fig 6.

### Foot morphology, digital deformity and foot disease outcomes

Due to a relatively small sample size, there was not enough data to conduct Chi^2^ tests for each category of the variables. For the Chi^2^ tests, expected cell counts were not more than five for the following variables: over-riding digits, forefoot amputation, flexible flatfoot (not enough instances), hyperkeratosis and history of ulceration (too many instances). There were no relationships found between the presence of digital deformity and foot morphology, nor the presence of deformity with foot disease (P>0.05 all).

Table 6 provides the summary of the gender-specific demography data, which forms the baseline information for a typical population from this group, and that data can be used for other relevant research purposes.

**Table 6:** Gender-specific demography data used in the clinical audit.

	Typical males in this study population	The typical female in this study population
Total (n, %)	43, 61.40%	27, 38.60%
Age (mean, median)	64.09, 63	65.62, 67
BMI (Avg)	31.20	30.70
Duration of diabetes (Avg)	14.34	13.66
Funding source/provider	Closing the Gap, n = 3	Enable NSW, n = 23
	Enable NSW, n = 32	Privately funded, n = 2
	Privately funded, n = 5	Private health fund, n = 1
	Private health fund, n = 1	NDIS, n = 1
	Aged care package, n = 1	
	NDIS, n = 1	

## Discussion

The purpose of the retrospective clinical audit was to capture the demographic, medical and foot-related characteristics of people with diabetes and neuropathy who present to pedorthic services at risk of plantar forefoot ulceration and to understand the referral pathways used to arrive at the service. In the absence of any previous data on a pedorthic patient population, this study aimed to understand better the population requiring therapeutic footwear to prevent diabetes-related neuropathic plantar forefoot ulcer occurrence and recurrence and how they access these services.

This study shows more male patients than female patients presented to the pedorthic clinic for treatment, but health-specific population study data (general population, not specific to diabetes) [26–28] represent an almost 50/50 ratio of males and females in Australia. Our data were consistent with previous study recruitment gender distributions in those with diabetes and at risk of related foot disease [4, 29]. This study also showed that the majority of the participants (n = 42) were born outside of Australia, and this is well aligned with a recent Australian epidemiological study data [1]. These demographic characteristics have implications for the provision of services as footwear and foot care practices vary widely by culture and gender.

A large number of people presenting to these clinics had overweight/obesity, rheumatoid arthritis, peripheral vascular disease and lymphodema [30]. This is expected in a population with a history of diabetic plantar ulceration and highlights the complexity and variability of the cohort. This population are highly likely to be accessing multiple services and require multidisciplinary care even beyond that required for diabetes care. These comorbidities also have implications for pedorthic interventions as the resulting lower limb functional changes require accommodation in footwear [19].

The foot morphology of those accessing pedorthics services varied substantially and included a large proportion of people with flatfoot deformity not often associated with plantar forefoot ulceration. This highlights the need for individual assessment when managing the footwear needs of those vulnerable to plantar foot ulceration.

A range of funding pathways were utilised to access the service, including state-based means-tested funding and disease and age-dependent funding. A small proportion of people accessed the service using private funds.

It was expected that associations among comorbidities and foot characteristics would be evident in the data due to current understandings of causative factors. However, these associations were not found in the data. It is likely that our sample was underpowered to detect these relationships due to the size of the sample.

There are some other limitations of this retrospective clinical audit. Generalisability may be limited since a single clinic in a defined catchment was audited. While this has its limitations, there is so little data available on those who access these services; the insights are still valuable for an initial understanding of this population. The majority of the participants were from the same region with a population of diverse cultural backgrounds. This captures a majority of common Australian patient types that are seen in high-risk foot services in metropolitan areas. The primary referrals to this clinic come from the high-risk foot services at hospitals in various regions across the city, catering to a diverse demographic.

Additionally, as a clinical audit, data were not measured prospectively and systematically but rather recorded from clinical note-keeping, limiting the reliability and validity of this approach. However, given the lack of data on who accesses these services, the study provides a basis for further research.

## Conclusion

In conclusion, this study aimed to profile patients accessing pedorthic services to prevent plantar forefoot ulceration. The retrospective clinical audit yielded baseline sociodemographic, foot pathology, and comorbidity-related data, along with insights into funding criteria and common practice trends. Understanding patient characteristics can inform healthcare strategies for preventive care, potentially reducing diabetes-related neuropathic foot ulcers and improving overall health. Further research is needed to establish effective multidisciplinary care approaches between medical professionals, podiatrists, and pedorthists for optimised patient outcomes.

## Supporting information

S1 File Clinical audit tool. (DOCX)

S2 File Clinical audit questionnaire and database. (XLSX)
