# Supplementary material for: Examining characteristics of those who receive pedorthic services: A clinical audit
Source: PLoS One. 2024 Jul 1;19(7):e0304443. doi: 10.1371/journal.pone.0304443 (PMC11216586; doi:10.1371/journal.pone.0304443)
Supplement: S1 File — (DOCX) [file pone.0304443.s001.docx]

## Supplementary Material 1 - Clinical audit tool

| **Clinical audit questionnaire and database** | | | | | | | | | | | | | | | | | | | | | | | | | | | | | | | | | | |
| --- | --- | --- | --- | --- | --- | --- | --- | --- | --- | --- | --- | --- | --- | --- | --- | --- | --- | --- | --- | --- | --- | --- | --- | --- | --- | --- | --- | --- | --- | --- | --- | --- | --- | --- |
| **Version 1** | | | | | | | | | | | | | | | | | | | | | | | | | | | | | | | | |  | |
| Sl# | Age (Years) | Sex | Country of Birth | Aboriginal/Torres Strait Islander? | Postcode | Duration of diabetes | Duration of neuropathy | Type of diabetes | | Height (cm) | Body Weight (kg) | Forefoot Pathology | | | | | | | | | | | | | Comorbidities | | | | | | | Footwear Fund provider | |  |
|  |  |  |  |  |  |  |  | T1DM | T2DM |  |  | Neuropathy | HAV | Hammer toe | Clawed toe | Overriding digits | Forefoot amputation | Partial amputation | Forefoot ulcers | Bony prominence | Flexible flat foot | Rigid flatfoot | LJM | Cavus foot | hyper keratosis | RA | PVD/PAD | Lymphodema | PTD | Altered foot posture | History of Ulceration |  | |  |
|  |  |  |  |  |  |  |  |  |  |  |  |  |  |  |  |  |  |  |  |  |  |  |  |  |  |  |  |  |  |  |  |  | |  |
|  |  |  |  |  |  |  |  |  |  |  |  |  |  |  |  |  |  |  |  |  |  |  |  |  |  |  |  |  |  |  |  |  | |  |
